# A novel *f*-divergence based generative adversarial imputation method for scRNA-seq data analysis

**DOI:** 10.1371/journal.pone.0292792

**Published:** 2023-11-10

**Authors:** Tong Si, Zackary Hopkins, John Yanev, Jie Hou, Haijun Gong

**Affiliations:** 1 Department of Mathematics and Statistics, Saint Louis University, St. Louis, MO, United States of America; 2 Department of Computer Science, Saint Louis University, St. Louis, MO, United States of America; IBM Research - Israel, ISRAEL

## Abstract

Comprehensive analysis of single-cell RNA sequencing (scRNA-seq) data can enhance our understanding of cellular diversity and aid in the development of personalized therapies for individuals. The abundance of missing values, known as dropouts, makes the analysis of scRNA-seq data a challenging task. Most traditional methods made assumptions about specific distributions for missing values, which limit their capability to capture the intricacy of high-dimensional scRNA-seq data. Moreover, the imputation performance of traditional methods decreases with higher missing rates. We propose a novel *f*-divergence based generative adversarial imputation method, called sc-*f*GAIN, for the scRNA-seq data imputation. Our studies identify four *f*-divergence functions, namely cross-entropy, Kullback-Leibler (KL), reverse KL, and Jensen-Shannon, that can be effectively integrated with the generative adversarial imputation network to generate imputed values without any assumptions, and mathematically prove that the distribution of imputed data using sc-*f*GAIN algorithm is same as the distribution of original data. Real scRNA-seq data analysis has shown that, compared to many traditional methods, the imputed values generated by sc-*f*GAIN algorithm have a smaller root-mean-square error, and it is robust to varying missing rates, moreover, it can reduce imputation variability. The flexibility offered by the *f*-divergence allows the sc-*f*GAIN method to accommodate various types of data, making it a more universal approach for imputing missing values of scRNA-seq data.

## Introduction

The genomics and transcriptomics studies have been revolutionized by the swift advancements in single-cell RNA sequencing (scRNA-seq) technology, which enable researchers to simultaneously profile the transcriptomes of thousands of individual cells [1, 2], providing a comprehensive view of the cellular heterogeneity within a tissue or organism [3]. Most of studies [4–9] are based on the traditional bulk RNA-seq or microarray experiments, which calculate the mean gene expression profile of cells in a sample, ignoring the heterogeneity and genomic variability among individual cells. Clinical studies have found that many drugs are not effective to some patients due to the cellular diversity or heterogeneous effects across individuals [10]. The scRNA-seq data can help identify distinct cell types that may have different functions or respond differently to the same stimuli or treatment [11]. scRNA-seq data can also be used to reconstruct cell-type-specific regulatory networks [12] and identify important regulatory processes or patterns that correspond to specific cell types or processes. Comprehensive analysis of scRNA-seq data can improve our understanding of cellular heterogeneity and disease mechanisms, and potentially help develop personalized and targeted treatments for individual patients based on their unique cellular profiles [13].

The missing values, which account for more than 50% and sometimes over 90% of the scRNA-seq data [14, 15], are often represented as 0s or values close to 0, making the scRNA-seq data analysis a challenging task [16]. Prevalence of missing values causes inaccurate reconstruction of cell-type-specific networks, consequently limits the potential power and benefits of single-cell RNA sequencing technologies [17, 18]. For instance, the missing data could get confounded with some genuinely low captured data, leading to a failure in identifying the regulatory functions [16] and deciphering the underlying biological mechanisms of specific cells.

Numerous computational techniques [19–25] have been developed to impute the missing values in the scRNA-seq data. Although some methods have shown efficacy in recovering missing values in small-scale scRNA-seq data, each method faces different challenges [26]. Markov Affinity-based Graph Imputation of Cells (MAGIC) [21] method creates a Markov transition matrix using the similarity matrix of single cells described by the Pearson correlation or mutual information to impute missing values. However, the MAGIC method necessitates the use of information from analogous cells or the consolidation of genes derived from scRNA-seq data that has been observed. This can result in inaccurate estimation of cell variability and reduced gene ranking performance [26], as well as potential oversmoothing of the data. SAVER [22] introduces different prior distributions to model the observed data, then develops a Bayesian-based model to impute missing values for each cell. Due to its dependence on a Markov Chain Monte Carlo algorithm for adjusting all parameters, its computational expenses are significant and may render it unscalable for extensive datasets [27]. PBLR [23] is a bounded low-rank recovery imputation method that constructs a consensus matrix and employs hierarchical clustering to identify cell sub-populations and submatrices for imputation. Another popular method, scImpute [14] assumes majority of the genetic information can be captured by a smaller number of latent factors, and utilizes an autoencoder to approximate the absent values. Most of these traditional methods [20, 22, 23, 28, 29] assume the missing values follow some specific distributions, so they could not accurately capture the intricacy of the high-dimensional scRNA-seq data. Furthermore, the imputation accuracy of these methods will decrease as the missing rate increases.

The outstanding performance of deep learning methods have attracted lot of research attention in the field of computational biology [24]. Deep generative methods, such as generative adversarial networks (GANs) [30], have emerged as powerful techniques for learning models from real-world data [31, 32]. GANs have demonstrated exceptional proficiency in diverse domains such as generating images [30] and videos [33], as well as image inpainting, where the missing parts of a corrupted image are filled in based on the remaining portion with outstanding performance. Recently, a new imputation method called GAIN, also known as generative adversarial imputation nets [34], was proposed to recover missing values. GAIN uses a vanilla GAN architecture with a generator network to impute missing values while a discriminator network evaluates the quality of the imputed values. GAIN has shown promising results in imputing missing values with an adversarial loss described by a binary cross entropy (BCE) function. However, there are still some limitations associated with this method. For instance, the use of the cross-entropy loss function may be inadequate for some intricate distributions. Moreover, the original GAN architecture that GAIN used has a serious problem called mode collapse. One possible solution to address these problems is to incorporate *f*-divergence in the GAN’s architecture.

In this work, we are motivated to develop a novel imputation approach that does not rely on distribution assumptions, called sc-*f*GAIN, standing for *f*-divergence based generative adversarial imputation network, for the scRNA-seq data analysis. Our work has two major novelties compared with vanilla GAIN and other existing methods. For the first time, our studies identify four *f*-divergence functions, namely cross-entropy, Kullback-Leibler (KL), reverse KL, and Jensen-Shannon, that can be integrated with GAIN to generate imputed values without any assumptions, and mathematically prove that the distribution of imputed data using sc-*f*GAIN algorithm is same as the distribution of original data. Finally, we evaluated the effectiveness and potential limitations of sc-*f*GAIN method by training it on real scRNA-seq data and comparing it to other imputation methods.

## Materials and methods

Generative modeling is a technique to learn the underlying probability distribution of a high-dimensional real-world dataset. Generative adversarial networks (GANs) are a class of deep generative models which use two competing neural networks to produce new data that resembles the real-world training data which follows an unknown probability distribution (*x* ∼ *p*(*x*)) [30]. These two networks are called Generator (*G*(*θ*)) and Discriminator (*D*(*ϕ*)), which are implemented as deep neural networks. The objective of the Generator is to transform a noise vector (*z* ∼ *q*(*z*)) to generate synthetic/fabricated samples (*G*(*z*, *θ*)) that are indistinguishable from authentic samples to trick the Discriminator. On the contrary, the role of the Discriminator is to differentiate the authentic samples (*x*) from the fabricated ones (*G*(*z*, *θ*)) produced by the Generator. *θ* and *ϕ* are the parameters of the Generator and Discriminator networks, respectively, and they are learned using Backpropagation algorithm by minimizing the cross entropy loss functions in the vanilla GANs [30]. The primary objective of the vanilla GAN is to minimize the generator loss, meanwhile maximize the discriminator loss, which is equivalent to solving the minimax optimization problem:

$$\begin{array}{c} \underset{G}{\text{min}}\;\underset{D}{\text{max}}\;L\left(D,G\right)=\underset{\theta }{\text{min}}\;\underset{\phi }{\text{max}}[E_{x}\left(\text{log}\;D\left(x,\phi \right)\right)+E_{z}\left(\text{log}\left(1-D\left(G\left(z,\theta \right),\phi \right)\right)\right)], \end{array}$$ (1)

Model collapse is a serious problem in the vanilla GANs where the generator network produces limited variations of samples that do not capture the diversity of the true data distribution, resulting in the discriminator network becoming too skilled at discriminating them [35]. One reason for mode collapse in vanilla GANs is the binary cross-entropy loss, which is not suitable for some complex distributions and might not capture the high-dimensional structure of the data. One possible solution to address this problem is to replace cross-entropy loss by the *f*-divergence based loss function to mitigate the mode collapse problem in GANs. *f*-divergence is a family of statistical measures which quantify the discrepancy between two distributions, with a higher value indicating greater dissimilarity between the generated samples and the actual data. Next we will briefly introduce the *f*-GAN [36] method.

### *f*-GAN: *f*-divergence based generative adversarial networks

*f*-GAN, or the *f*-divergence based generative adversarial network, is a variant of generative adversarial networks with the loss function change to the class of *f*-divergence, which is a generalization of Kullback-Leibler (KL) divergence and includes other divergences such as reverse KL, Jensen-Shannon as special cases. The *f*-GAN framework was first proposed in Nowozin *et al*.’s work [36] to generate synthetic pictures. To provide a better understanding of this framework, we will briefly review some fundamental concepts related to *f*-divergence and *f*-GAN.

The *f*-divergence, also known as the Ali-Silvey distances [37], between two probability distributions *P* and *Q* is defined as:

$$\begin{array}{c} D_{f}\left(P||Q\right)=E_{x∼q}[f(\frac{p(x)}{q(x)})]=\int_{\Omega }q\left(x\right)f(\frac{p(x)}{q(x)})dx, \end{array}$$ (2)

where, *p*(*x*) and *q*(*x*) are two continuous probability density function on the domain Ω, and the generator function *f* is a proper, lower-semicontinuous, convex generator function with *f*(1) = 0. Since the *f*-divergence measures the difference between two distributions, so, it could be used as a measure of the loss functions in the GANs.

The estimation of *f*-divergence need use the convex conjugate of a convex function. Every convex lower-semicontinuous function *f* has a convex conjugate function f*, which is also known as Fenchel-Legendre transform of *f*. The convex conjugate is defined to be [38]:

$$\begin{array}{c} f^{*}\left(t\right)=\underset{u\in {\text{dom}}_{f}}{\text{sup}}\left\{ut-f\left(u\right)\right\}. \end{array}$$ (3)

It has been proved that f* is also convext and lower-semicontinuous if *f*(*x*) is a convex function on R. The lower bound of the *f*-divergence can be estimated using the Fenchel conjugate function, which is expressed as [36]:

$$\begin{array}{cc} D_{f}\left(P||Q\right) & =\int_{\Omega }q\left(x\right)\;\underset{T\in T}{\text{sup}}\;\{t\frac{p(x)}{q(x)}-f^{*}\left(t\right)\}dx\\ & \geq \underset{T\in T}{\text{sup}}\;(\int_{\Omega }(p\left(x\right)T\left(x\right)-q\left(x\right)f^{*}(T\left(x\right))dx)\\ & =\underset{T\in T}{\text{sup}}\;(E_{x∼P}\left[T\left(x\right)\right]-E_{x∼Q}\left[f^{*}\left(T\left(x\right)\right)\right]), \end{array}$$

where, *T*(*x*) is any Borel function. After taking the variation with respect to *T*, we get $T^{*}\left(x\right)=f^{\prime }\left(\frac{p(x)}{q(x)}\right)$ which can be used to choose the generator function *f* and design the class of functions *T*.

Given a convex function *f*, the objective of *f*-GAN is to minimize the *f*-divergence *D*_*f*_(*P*||*Q*), that is, solve the following minimax problem using the variational divergence minimization (VDM) method [36]:

$$\begin{array}{c} \underset{Q}{\text{min}}\;\underset{T}{\text{sup}}(E_{x∼P}\left[T\left(x,\phi \right)\right]-E_{x∼Q(z,\theta )}\left[f^{*}\left(T\left(x\right)\right)\right]), \end{array}$$ (4)

where, *Q*(*z*, *θ*) is a generator implemented by a neural network, which takes a random vector *z* as input and output a sample of interest. The Borel function *T*(*x*) is called a critic function, which is also approximated by a neural network. If *S*_*ϕ*_ is the output of neural network with parameters *ϕ*, *g*_*f*_ is an output activation function specific to the choice of *f*-divergence, then we can have the critic function being clarified as *T*(*x*) = *g*_*f*_(*S*_*ϕ*_(*x*)), and the above minimax problem can be expressed as

$$\begin{array}{c} \underset{\theta }{\text{min}}\;\underset{\phi }{\text{sup}}(E_{x∼P}\left[g_{f}\left(S_{\phi }\left(x\right)\right)\right]-E_{x∼Q(z,\theta )}\left[f^{*}\left(g_{f}\left(S_{\phi }\left(x\right)\right)\right)\right]). \end{array}$$ (5)

By using a variety of *f* functions and output activation functions *g*_*f*_, we can obtain various *f*-divergence [36], including the divergence of cross entropy (CE), Kullback-Leibler (FKL), Reverse KL (RKL), Jensen-Shannon (JS), Pearson *χ*^2^ (PC), etc. In the next section, we provide a theoretical proof that only four *f*-divergence based loss functions can be effectively utilized for the imputation of single-cell RNA sequencing data. The *f*-divergences are more general and computationally efficient, and can be used in different GAN architectures to offer a flexible and efficient way to measure the distance between probability distributions. Next, we will discuss our sc-*f*GAIN method, *f*-divergence based generative adversarial imputation network, for the imputation of missing values in the scRNA-seq data.

### sc-*f*GAIN: *f*-divergence based generative adversarial imputation network

**Fig 1:**
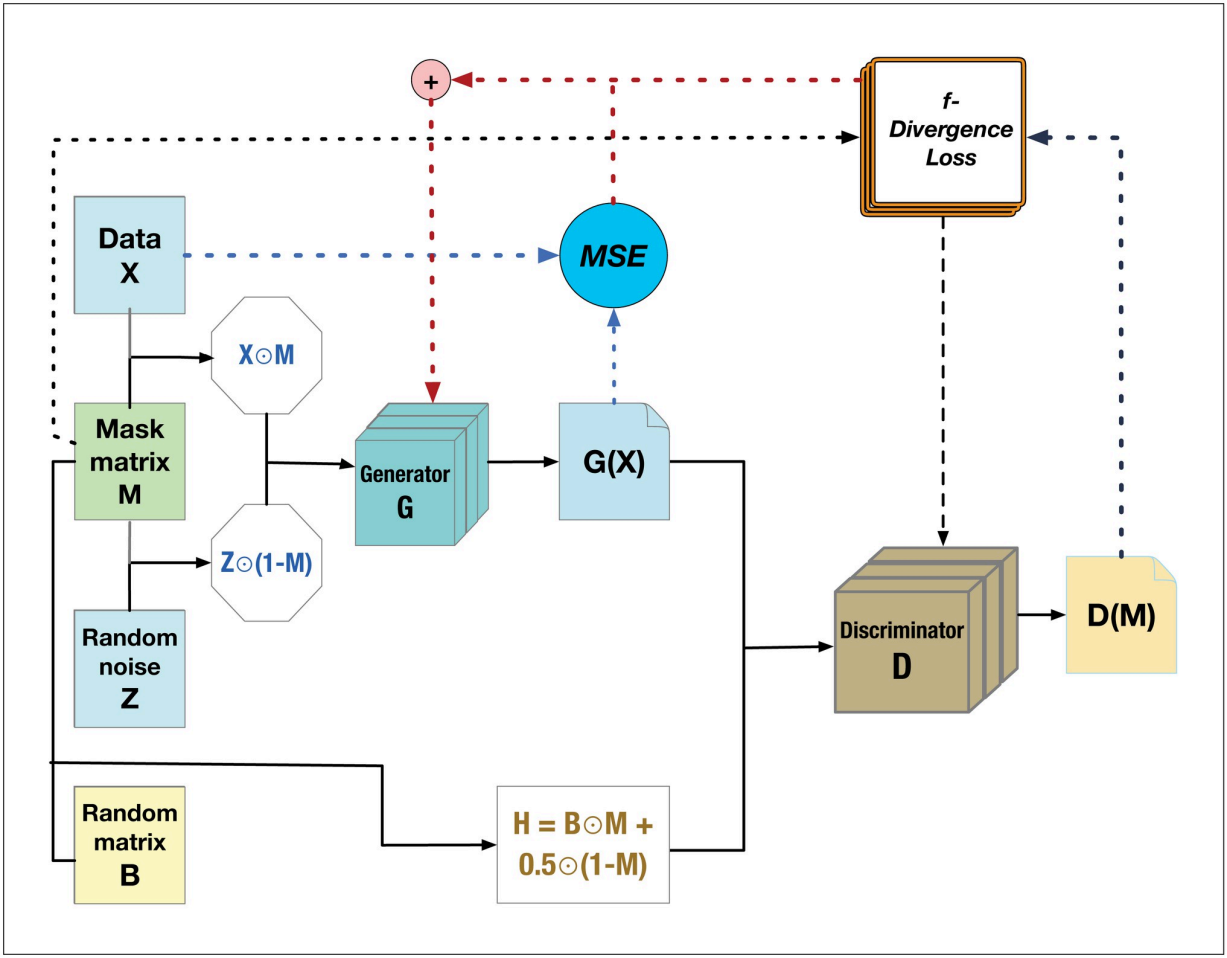
An illustration of the scRNA-seq f-divergence based generative adversarial imputation network (sc-*f*GAIN) architecture. The generator takes as input the incomplete scRNA-seq data and a corresponding mask matrix, along with a random matrix to generate synthetic imputed data. The discriminator distinguishes between real and imputed values generated by the generator. Both generator and discriminator are trained using *f*-divergence loss functions.

The generative adversarial imputation nets (GAIN) method [34] was proposed to recover missing values implemented by a vanilla GAN, which uses binary cross-entropy (CE) loss to train models. Sometimes the cross-entropy divergence is not suitable for some complex distributions of scRNA-seq data. The sc-*f*GAIN algorithm is proposed to integrate *f*-divergence with GAIN model to build a more universal and efficient imputation architecture. Fig 1 illustrates the workflow of our sc-*f*GAIN method to impute missing values of scRNA-seq data, and the theoretical proof is provided in the next section.

#### Generator and discriminator

We adopt the notations used in Yoon *et al*.’s work [34] for clarity and coherence. The data vector **X** = (*X*_1_, …, *X*_*d*_) measures the expression levels of *d* genes and may contain both observed and missing values. To identify which components are missing or observed, a mask vector **M** = (*M*_1_, …, *M*_*d*_) is introduced, where *M*_*i*_ ∈ {0, 1} denotes whether the *i*-th component is observed (*M*_*i*_ = 1) or missing (*M*_*i*_ = 0).

One key component of the sc-*f*GAIN architecture is Generator *G*, which is implemented using convolutional neural networks. The Generator takes the sc-RNAseq dataset **X**, random noise vector **Z**, and binary mask **M** as inputs and generates imputed values for the missing observations. The input can be represented as the product of the element-wise multiplication between the mask matrix **M** and the raw data matrix **X**, and the element-wise multiplication between the complement of mask matrix, (**1** − **M**) and random noise matrix **Z**: **M** ⊙ **X** + (**1** − **M**) ⊙ **Z**, where the symbol ⊙ represents the Hadamard product or element-wise multiplication between two matrices of the same dimensions. The output of the Generator, denoted as *G*(**X**, **M**, **Z**), will be used to fill in the dropouts in the observed data **X** using the non-missing entries of **X** and the noise vector **Z**. The complete data, represented by $\hat{\text{X}}$, will be used as input to the discriminator. It comprises of the combination of imputed values *G*(**X**, **M**, **Z**) generated by *G* and the observed elements of **X**, which is expressed as

$$\begin{array}{c} \hat{\text{X}}=\text{M}⊙\text{X}+\left(1-\text{M}\right)⊙G\left(\text{X},\text{M},\text{Z}\right). \end{array}$$ (6)

The second key component of the sc-*f*GAIN architecture is Discriminator *D*, which acts as an adversary or judge. Both the observed data and imputed values produced by the Generator network are input to the Discriminator network. The Discriminator’s role is to distinguish between real (observed) data and imputed data, thereby predict the binary mask vector **M**. Theorem 1–2 in the next section identified four *f*-divergence functionss (including CE, FKL, RKL, and JS), and proved that, given a fixed generator, there always exists one optimal discriminator; and given an optimal discriminator, we can always obtain optimal generators. In order to obtain a unique optimal solution for the Generator network *G*, Theorem 3 and Yoon’s work [34] have proved that the input to the Discriminator should not only consist of generated “imputed” values and observed data, but also some additional information, such as the hint matrix **H**, which should be designed to contain adequate information about the binary mask vector **M** in order to facilitate the training of the Discriminator *D*. Yoon *et al*’s work [34] introduced a binary random variable **B** = (*B*_1_, …, *B*_*d*_) ∈ {0, 1}^*d*^, which indicates the location of missing values in the input data. To determine the value of *B*_*j*_ in the equation, a random value *k* is sampled uniformly from the set 1, …, *d*, then, *B*_*j*_ = 1 if *j* ≠ *k*, and *B*_*j*_ = 0 otherwise. If **B** and **M** are independent, according to [34], hint matrix is expressed as:

$$\begin{array}{c} \text{H}=\text{B}⊙\text{M}+0.5(1-\text{B}). \end{array}$$ (7)

Theorem 4 proves that if the hint variable **H** is sampled using Eq 7, the generator has the capability to replicate the desired distribution of the data, resulting in a unique distribution. The proof in Yoon *et al*’s work [34] assumes the binary cross-entropy adversarial loss. Our theoretical studies in the next section prove that this finding holds true if we use four different *f*-divergence functions for the adversarial loss.

#### *f*-divergence based objective functions

The objective of sc-*f*GAIN is to impute missing values by training a generative model to learn to produce realistic data samples from some incomplete dataset. To achieve this, the objective functions, which serve as a measure of how well the generator and discriminator are performing their respective tasks, will be used to guide the optimization process towards better imputation results. Specifically, the generator network is trained to reduce the difference between the imputed data and real data, whereas the discriminator network is trained to differentiate between the imputed data and real data.

The input of discriminator includes the “complete” data $\hat{\text{X}}$ described by the Eq 6, which combines both imputed values *G*(**X**, **M**, **Z**) generated by *G* and the observed elements of **X**, and hint matrix **H**. The output of the discriminator is $\hat{\text{M}}=D\left(\hat{\text{X}},\text{H}\right)$, given the mask matrix **M**, the vanilla GAIN [34]’s objective function for the discriminator *D* is described by a cross entropy function:

$$\begin{array}{c} L_{DG}\left(\hat{\text{X}},\text{M},\text{H}\right)=E_{\hat{X},M,H}\left[{\text{M}}^{T}\;\text{log}\;D\left(\hat{\text{X}},\text{H}\right)+{\left(1-\text{M}\right)}^{T}\;\text{log}\left(1-D\left(\hat{\text{X}},\text{H}\right)\right)\right]. \end{array}$$

Now, we generalize the objective function of cross entropy to *f*-divergence, given the output of the network $S_{\phi }\left(\hat{\text{X}},\text{H}\right)$ and output activation function *g*_*f*_, we can rewrite the Eq 5 and get the *f*-divergence based objective function for the discriminator in the sc-*f*GAIN architecture:

$$L_{DG,f}\left(\hat{\text{X}},\text{M},\text{H}\right)=E_{x∼P}\left[g_{f}\left(S_{\phi }\left(\hat{\text{X}},\text{H}\right)\right)\right]-E_{x∼Q(z,\theta )}\left[f^{*}\left(g_{f}\left(S_{\phi }\left(\hat{\text{X}},\text{H}\right)\right)\right)\right]$$ (8)

$$=E_{\hat{X},M,H}\left[{\text{M}}^{T}g_{f}\left(S_{\phi }\left(\hat{\text{X}},\text{H}\right)\right)-{\left(1-\text{M}\right)}^{T}f^{*}\left(g_{f}\left(S_{\phi }\left(\hat{\text{X}},\text{H}\right)\right)\right)\right].$$ (9)

To train the discriminator *D*, we first fix the generator *G* and sample mini-batches of size *k*_*D*_ from the dataset, then, we optimize the discriminator by minimizing the loss function $L_{DG,f}$ over the mini-batches: $\underset{D}{\text{min}}-\sum_{j=1}^{k_{D}}L_{DG,f}\left(\hat{\text{x}}\left(j\right),\text{m}\left(j\right),\text{h}\left(j\right)\right)$. Table 1 summarizes five objective functions of discriminator based on different *f*-divergence functions and activation functions *g*_*f*_, where the sigmoid function $D_{\phi }\left(x\right)=\frac{1}{1+{\text{exp}}^{-S_{\phi }\left(x\right)}}$ is applied on the output of the discriminator network *S*_*ϕ*_(*x*). The abbreviations CE, FKL, RKL, JS, and PC will be used to represent the divergence of cross entropy, forward KL, reverse KL, Jensen-Shannon, and Pearson *χ*^2^ respectively.

**Table 1:** Objective functions of the discriminator in the sc-*f*GAIN models based on different *f*-divergence function.

Divergence	Objective function
CE	$L_{D}=E_{\hat{X},M,H}{\text{M}}^{T}(\text{log}\;D(\hat{\text{X}},\text{H}))+{\left(1-\text{M}\right)}^{T}(\text{log}\;\left(1-D\left(\hat{\text{X}},\text{H}\right)\right))$
FKL	$L_{D}=E_{\hat{X},M,H}{\text{M}}^{T}(\text{log}\;\frac{D(\hat{\text{X}},\text{H})}{1-D(\hat{\text{X}},\text{H})})+{\left(1-\text{M}\right)}^{T}(-\frac{D(\hat{\text{X}},\text{H})}{e(1-D\left(\hat{\text{X}},\text{H}\right))})$
RKL	$L_{D}=E_{\hat{X},M,H}{\text{M}}^{T}(1-\frac{1}{D(\hat{\text{X}},\text{H})})+{\left(1-\text{M}\right)}^{T}(\text{log}\;\frac{e(1-D\left(\hat{\text{X}},\text{H}\right))}{D(\hat{\text{X}},\text{H})})$
JS	$L_{D}=E_{\hat{X},M,H}{\text{M}}^{T}(\text{log}\;2D(\hat{\text{X}},\text{H}))+{\left(1-\text{M}\right)}^{T}(\text{log}\;\left(2-2D\left(\hat{\text{X}},\text{H}\right)\right))$
PC	$\begin{array}{cc} & L_{D}=E_{\hat{X},M,H}{\text{M}}^{T}(-\text{log}\frac{1-D(\hat{\text{X}},\text{H})}{D(\hat{\text{X}},\text{H})})\\ & \;+{\left(1-\text{M}\right)}^{T}(-\frac{1}{4}{\left(\text{log}\frac{1-D(\hat{\text{X}},\text{H})}{D(\hat{\text{X}},\text{H}}\right)}^{2}+\text{log}\frac{1-D(\hat{\text{X}},\text{H})}{D(\hat{\text{X}},\text{H})}) \end{array}$

After the discriminator *D* is updated, the next step is to optimize the generator *G*. The generator’s optimization is dependent on two loss functions: reconstruction loss $L_{R}$ for the observed entries (*m* = 1) and adversarial loss $L_{GA,f}$ for the missing entries (*m* = 0). Similar to the original GAIN [34], we use the mean squared error (MSE) to calculate the sc-*f*GAIN’s reconstruction loss between the generated data and the original data: $L_{R}\left(\text{X},{\text{X}}^{\prime }\right)=||\text{X}-{\text{X}}^{\prime }{\left|\right|}^{2}$, where, ||⋅|| is the Euclidean norm, and **X**′ = *G*(**X**, **M**, **Z**) is imputed value matrix given the observed value matrix **X**. Minimizing the reconstruction loss $L_{R}$ ensures that the generated values for the observed entries (*m* = 1) are as close as possible to the original values. Minimizing the adversarial loss $L_{GA,f}$ will train the generator to generate “realistic” imputed values for the missing elements (*m* = 0) that are difficult for the discriminator to distinguish from real values. The *f*-divergence based adversarial loss for the generator $L_{GA,f}$ can be derived by setting **M** = 0 in the Eq 9:

$$\begin{array}{c} L_{GA,f}\left(\hat{\text{X}},\text{M},\text{H}\right)=-E_{\hat{X},M,H}{\left(1-\text{M}\right)}^{T}f^{*}\left(g_{f}\left(S_{\phi }\left(\hat{\text{X}},\text{H}\right)\right)\right). \end{array}$$ (10)

The overall objective function $L_{Gf}$ for the generator can be written as a weighted sum of these two loss functions, where the weight λ is a hyperparameter that need to be tuned to balance between the reconstruction accuracy and the data distribution fidelity for better imputation performance:

$$\begin{array}{c} L_{G,f}=L_{GA,f}\left(\hat{\text{X}},\text{M},\text{H}\right)+\lambda L_{R}\left(\text{X},{\text{X}}^{\prime }\right). \end{array}$$ (11)

Algorithm 1 presents the pseudo-code for the sc-*f*GAIN method, which employs different *f*-divergences, instead of the cross entropy used in the original GAIN [34], to quantify the difference between two distributions. The sc-*f*GAIN imputation process involves training both generator and discriminator in order to minimize the *f*-divergence based loss functions defined by Eqs 9 and 11, respectively. The replacement of cross entropy in the loss function by the *f*-divergence is a non-trivial task since some divergence functions can not be used for the GAIN’s imputation procedure. In the next section, we will perform a theoretical analysis of our sc-*f*GAIN algorithm.

**Algorithm 1** Pseudo-code of sc-*f*GAIN

**Input**: Single-cell RNAseq Data **X**, Mask matrix **M**, *f*-divergence functions

**Initialization**

Define hyperparameters, number of iterations, batch size, etc

Initialize network parameters

**Discriminator and Generator optimization**

**While** training loss has not converged

Draw minibatches of *k*_*D*_ samples of {**x**} from **X**

Draw minibatches of *k*_*D*_ samples of {**z**} from **Z**

Draw minibatches of *k*_*D*_ samples of {**b**} from **B**

Update the following matrix

**X**′ = *G*(**X**, **M**, **Z**)

$\hat{\text{X}}=\text{M}⊙\text{X}+\left(1-\text{M}\right)⊙{\text{X}}^{\prime }$

**H** = **B** ⊙ **M** + 0.5(1 − **B**)

$\hat{\text{M}}=D\left(\hat{\text{X}},\text{H}\right)$

For a fixed *G*, **Update** *D* using stochastic gradient descent (SGD) method for each sample

$\nabla_{D}-\sum_{j=1}^{k_{D}}L_{DG,f}\left(\hat{\text{x}}\left(j\right),\text{m}\left(j\right),\text{h}\left(j\right)\right)$

For a fixed *D*, **Update** *G* using SGD method for each sample

$\nabla_{G}\sum_{j=1}^{k_{G}}L_{GA,f}\left(\hat{\text{x}}\left(\text{j}\right),\text{m}\left(\text{j}\right),\text{h}\left(\text{j}\right)\right)+\lambda L_{R}\left(\text{x}\left(\text{j}\right),{\text{x}}^{\prime }\left(\text{j}\right)\right)$

**Output**: Complete data matrix

## Theoretical analysis of sc-*f*GAIN algorithm

In this section, we will identify specific *f*-divergence functions that can be used for the generative adversarial imputation network, and provide mathematical proof for the Algorithm 1. We adopt some notations and assumptions in Yoon *et al*’s work [34], and assume that **X** is independent of **M**, where *p*(**x**, **m**, **h**) denotes the joint distribution for the random variables $\left(\hat{\text{X}},\text{M},\text{H}\right)$, and $\hat{p}\left(\text{x}\right),p\left(\text{m}\right),p\left(\text{h}\right)$ are corresponding marginal distributions.

**Theorem 1**. *Let S*_*ϕ*_(**x**, **h**) *be a function*: χ→R, *where x* ∈ *χ*, h∈H *(hint space), and p*(**x**, **h**) > 0, *D be a function: χ* → [0, 1]^*d*^. *If the f-divergence based objective function is defined by the* Eq 9, then, given a fixed generator G, there always exists one optimal discriminator D*(**x**, **h**) *if f = CE, FKL, RKL, JS, PC*.

*Proof*. The *f*-divergence based objective function Eq 9 can be rewritten as

$$\begin{array}{cc} L_{DG,f}\left(\hat{\text{X}},\text{M},\text{H}\right) & =E_{\hat{X},M,H}\left[{\text{M}}^{T}g_{f}\left(S_{\phi }\left(\text{x},\text{h}\right)\right)-{\left(1-\text{M}\right)}^{T}f^{*}\left(g_{f}\left(S_{\phi }\left(\text{x},\text{h}\right)\right)\right)\right]\\ & =\int_{\chi }\int_{H}\sum_{i=1}^{d}g_{f}{\left(S_{\phi }\left(x,h\right)\right)}_{i}p\left(\text{x},\text{h},m_{i}=1\right)\\ & +f^{*}{\left(g_{f}\left(S_{\phi }\left(x,h\right)\right)\right)}_{i}p\left(\text{x},\text{h},m_{i}=0\right)dhdx. \end{array}$$

Given a fixed Generator *G*, the optimal Discriminator D* is obtained by solving the equation $\frac{\partial L_{DG,f}}{\partial S_{\phi }}=0$, that is

$$\begin{array}{c} \frac{\partial }{\partial g_{f}{\left(S_{\phi }\right)}_{i}}f^{*}{\left(g_{f}\left(S_{\phi }\right)\right)}_{i}=\frac{p(\text{x},\text{h},m_{i}=1)}{p(\text{x},\text{h},m_{i}=0)}. \end{array}$$ (12)

After inserting the *f*-divergence’s output activation functions and conjugate functions given in Table 2, and applying the sigmoid function $D_{\phi }\left(x\right)=\frac{1}{1+{\text{exp}}^{-S_{\phi }\left(x\right)}}$ on the output of the discriminator network *S*_*ϕ*_(*x*), we identified five *f*-divergences, including CE, FKL, RKL, JS, and PC, that always have an optimal discriminator D* given a fixed *G*, for *i* ∈ {0, 1}^*d*^,

$$\begin{array}{c} D^{*}{\left(\text{x},\text{h}\right)}_{i}=\{\begin{array}{cc} p(m_{i}=1|\text{x},\text{h}), & \text{if}\;f=\text{CE},\;\text{RKL},\;\text{JS}\\ \frac{p(m_{i}=1|\text{x},\text{h})e}{p\left(m_{i}=0|\text{x},\text{h}\right)+p\left(m_{i}=1|\text{x},\text{h}\right)e}, & \text{if}\;f=\text{FKL}\\ \frac{1}{exp(2-2p\left(m_{i}=1|\text{x},\text{h}\right)/p\left(m_{i}=0|\text{x},\text{h}\right))+1}, & \text{if}\;f=\text{PC}. \end{array} \end{array}$$

For a more detailed proof of Theorem 1, please refer to S1 Appendix.

**Table 2:** *f*-divergence’s output activation function, conjugate function, and the optimal discriminator D* for a given generator *G*, *p* = *p*(*x*, *h*, *m*_*i*_ = 1), and *q* = *p*(*x*, *h*, *m*_*i*_ = 0).

*f*-Divergence	Output activation *g*_*f*_(*s*)	Conjugate f*(*t*)	Optimal D*
CE	−log(1 + exp(−*s*))	−log(1 − exp(*t*))	$\frac{p}{p+q}$
FKL	*s*	exp(*t* − 1)	$\frac{pe}{pe+q}$
RKL	−exp(−*s*)	−1 − log(−*t*)	$\frac{p}{p+q}$
JS	log(2) − log(1 + exp(−*s*))	−log(2 − exp(*t*))	$\frac{p}{p+q}$
PC	*s*	$\frac{1}{4}t^{2}+t$	$\frac{exp(2(p-q)/q)}{1+exp(2(p-q)/q)}$

If we substitute the optimal discriminator D* derived in Theorem 1 into the objective function Eq 9, we obtain the loss function of the generator *G* as follows:

$$\begin{array}{c} L_{G,f}\left(D^{*}\right)=E_{\hat{X},M,H}\left[{\text{M}}^{T}g_{f}\left(S_{\phi }\left(D^{*}\right)\right)-{\left(1-\text{M}\right)}^{T}f^{*}\left(g_{f}\left(S_{\phi }\left(D^{*}\right)\right)\right)\right]. \end{array}$$ (13)

Then, by minimizing $L_{G,f}\left(D^{*}\right)$, we derived the second theorem.

**Theorem 2**. *The f-divergence based loss function* $L_{G,f}\left(D^{*}\right)$ *has a global minimum if and only if the density p satisfies*:

$$\hat{p}\left(\text{x}|\text{h},m_{i}=1\right)=\hat{p}\left(\text{x}|\text{h},m_{i}=0\right)=\hat{p}\left(\text{x}|\text{h}\right),$$ (14)

*for each i* ∈ {1, …, *d*}, *x* ∈ **X** *and* h∈H *such that p*(**h**|*m*_*i*_ = *t*) > 0. *And this theorem is true only if f = CE, FKL, RKL, JS*.

Yoon *et al*’s work [34] proved the validity of this theorem for the cross-entropy based loss function. We will prove that this theorem is also valid for the forward KL, reverse KL, and Jensen-Shannon divergence based loss functions described by Eq 13, but it does not hold for the Pearson *χ*^2^ divergence.

*Proof*. We will present a concise proof of this theorem, focusing on the KL-divergence case, which is more intricate compared to the cross-entropy scenario. After substituting D*, using the Eq 13 and objective function in the Table 1, the KL-divergence based loss function can be simplified as

$$\begin{array}{c} L_{G,f}\left(D^{*}\right)=\int_{\chi }\int_{H}\sum_{i=1}^{d}p\left(\text{x},\text{h},m_{i}=1\right)\;\text{log}\;\frac{p(\text{x},\text{h},m_{i}=1)}{p(\text{x},\text{h},m_{i}=0)}dhdx. \end{array}$$

It follows that if *p*(**x**, **h**, *m*_*i*_ = 1) = *p*(**x**, **h**, *m*_*i*_ = 0) for any *i* ∈ {1, …, *d*}, then $L_{G,f}\left(D^{*}\right)$ will be 0. The above loss function can also be rewritten as

$$\begin{array}{cc} L_{G,f}\left(D^{*}\right) & =\int_{\chi }\int_{H}\sum_{i=1}^{d}p\left(\text{x},\text{h},m_{i}=1\right)\left(\text{log}\;p\left(\text{x},\text{h},m_{i}=1\right)-\text{log}\;p\left(\text{x},\text{h},m_{i}=0\right)\right)dhdx\\ & =\sum_{t\in \{0,1\}}\sum_{i=1}^{d}\int_{H}p\left(\text{h},m_{i}=t\right)D_{KL}(p\left(\text{x}|\text{h},m_{i}=t\right)||p\left(\text{x}|\text{h}\right))dh\\ & +\sum_{i=1}^{d}\int_{H}p\left(h\right)D_{KL}(p\left(\text{x}|\text{h}\right)||p\left(\text{x}|\text{h},m_{i}=0\right))dh\\ & +\sum_{i=1}^{d}\int_{H}(\sum_{t\in \{0,1\}}p\left(\text{h},m_{i}=t\right)\text{log}\;p\left(m_{i}=t|\text{h}\right)-p\left(\text{h}\right)\text{log}\;p(m_{i}=0|\text{h}))dh. \end{array}$$

Since KL divergence *D*_*KL*_ is non-negative, so the loss function $L_{G,f}\left(D^{*}\right)$ is minimized if and only if $\hat{p}\left(\text{x}|\text{h},m_{i}=t\right)=\hat{p}\left(\text{x}|\text{h}\right)$ for any *i* ∈ {1, …, *d*}. The detailed proof for different *f*-divergence cases are given in the S1 Appendix.

In comparison to [34], our work in Theorem 1–2 offers a more general proof based on the *f*-divergence functions, establishing that the optimal discriminator and generator can be attained using the sc-*f*GAIN algorithm when the loss function is formulated using four distinct *f*-divergence functions: cross-entropy, KL, reverse KL, and JS divergence. Theorem 2 demonstrates the independence of **x** from the mask variable **M** given the hint variable **H**. The amount of information contained in **H** directly influences the learning capability of the generator *G*. If **H** contains less informative hints or lacks important information, the learning ability of the generator may be compromised, which is discussed in the Theorem 3.

**Theorem 3**. *In the sc-fGAIN algorithm, for f = CE, FKL, RKL, and JS, if the hint variable* **H** *is independent of mask variable* **M**, *then the density* $\hat{p}$ *in the Theorem 2 is not unique*.

*Proof*. Theorem 2 has proved that, $\hat{p}\left(\text{x}|\text{h},m_{i}=1\right)=\hat{p}\left(\text{x}|\text{h},m_{i}=0\right)=\hat{p}\left(\text{x}|\text{h}\right)$ is valid for *f* = CE, FKL, RKL, and JS. If **H** is independent of **M**, and **H** is conditionally independent of **X** given **M**, it is easy to verify that $\hat{p}\left(\text{x}|m_{i}=1\right)=\hat{p}\left(\text{x}|m_{i}=0\right)$, for all *i* ∈ {1, …, *d*}. Follow the same argumentation as [34] for the cross-entropy case, there are more parameters than the number of equations, so the density $\hat{p}$ is not unique.

To get a unique density solution, a hinting mechanism is needed such that **H** reveals some information of **M** to the discriminator *D*, which means that they are not independent. In the last section, we adopt the method proposed in [34] to sample the hint variable using the Eq 7, and assume **B** and **M** are independent. This hinting mechanism can ensure that the generator is capable of replicating the desired distribution of the data, that is the Theorem 4.

**Theorem 4**. *If the hint variable* **H** *is sampled according to* Eq 7, *then the density* $\hat{p}$ *in Theorem 2 is unique and satisfies* $\hat{p}\left(\text{x}|\text{m}\right)=\hat{p}\left(\text{x}|1\right)$ *for any vector* **m** ∈ {0, 1}^*d*^ *and f = CE, FKL, RKL, JS, where* $\hat{p}\left(\text{x}|1\right)$ *is the density of* **X**. *That is, the distribution of imputed data is same as the distribution of original data*.

*Proof*. The proof is similar to the CE scenario [34]. Theorem 2 has shown that $\hat{p}\left(\text{x}|\text{h},m_{i}=1\right)=\hat{p}\left(\text{x}|\text{h},m_{i}=0\right)$ holds for the *f*-divergence of CE, FKL, RKL and JS. Because of Eq 7, $\hat{p}\left(\text{x}|\text{h},m_{i}=1\right)=\hat{p}\left(\text{x}|b,m_{i}=1\right)=\hat{p}\left(\text{x}|\text{h},m_{i}=0\right)=\hat{p}\left(\text{x}|b,m_{i}=0\right)$ is valid. Since **B** and **M** are independent, it is easy to prove $\hat{p}\left(\text{x}|m_{i}=1\right)=\hat{p}\left(\text{x}|m_{i}=0\right)$. It means, for any two vectors **m**_**1**_, **m**_**2**_ ∈ {0, 1}^*d*^ that differ only on one component, we have $\hat{p}\left(\text{x}|{\text{m}}_{1}\right)=\hat{p}\left(\text{x}|{\text{m}}_{2}\right)$.

This equation also holds true for any two vectors **m**_**1**_ and **m**_**2**_ in {0, 1}^*d*^, because we can always find a sequence of vectors between **m**_**1**_ and **m**_**2**_, such that all the adjacent vectors differ from each other in only one component. Consequently, the imputed data distribution $\hat{p}\left(\text{x}|\text{m}\right)$ is the same for all possible vectors **m** ∈ {0, 1}^*d*^. This unique imputed data density, denoted by $\hat{p}\left(\text{x}|1\right)$, corresponds to the true data **X**’s density *p*(**x**), that is, $\hat{p}\left(\text{x}|\text{m}\right)=\hat{p}\left(\text{x}|1\right)=p\left(\text{x}\right)$. The proof is based on the Theorem 2, so it is true for *f* = CE, FKL, RKL, JS.

Theorem 1–4 theoretically confirm that the generative adversarial imputation network method remains valid if and only if the loss function is defined using four *f*-divergence, including CE, FKL, RKL, and JS divergence. The flexibility offered by the *f*-divergence formulation allows sc-*f*GAIN to accommodate various types of data and distributions, making it a more universal approach for imputing missing values.

## Results and discussion

In this section, we will implement the sc-*f*GAIN algorithm to impute missing values in single-cell RNA sequencing data. The sc-*f*GAIN algorithm leverages *f*-divergence and generative adversarial networks to generate imputations that can capture the complex dependencies within the data without relying on any assumptions. To evaluate the effectiveness of the sc-*f*GAIN algorithm, we compared its performance with that of other state-of-the-art imputation methods including MAGIC [21], scImpute [14], PBLR [23] and SAVER [22]. We designed several experiments to assess the performance of each method on the single-cell RNAseq data with varying missing rates, different metrics and setups. These experiments aim to determine the ability of each method to accurately impute missing values while preserving the underlying biological information.

### Data and configuration

In our experiments, we conducted an extensive evaluation of the effectiveness of our sc-*f*GAIN algorithm and various imputation techniques using real single-cell RNA sequencing (scRNA-seq) data derived from the UMI-based CellBench dataset GSE118767 [39], which was obtained through the 10x Chromium Genomics protocol [40]. As detailed in [39], this lung adenocarcinoma experimental design encompasses diverse scenarios such as single cells, mixtures of single cells, and mixtures of RNA from up to five distinct cancer cell lines (namely, H2228, H1975, A549, H838, and HCC827). In the RNA mixture experiment, the pseudo cells were created with varying amounts of input RNA, rendering it an ideal benchmark dataset for the assessment of normalization methods and the evaluation of imputation method performance. Comprising a total of 10,164 genes and 3,918 cells, this benchmark dataset provides an extensive transcriptional profile of mixed cell populations. It is widely recognized and frequently employed in contemporary scRNA-seq data analysis, serving as a robust foundation for our assessments. Furthermore, in our evaluation, we also incorporated ten bulk RNA-seq samples from GSE86337 [41]. This dataset contains two replicates for each of the five cell lines, providing additional resources for the comprehensive evaluation of various imputation methods.

Before analyzing scRNA-seq gene expression data, data processing is performed, including data filtering and quality control as the first step. Due to the high dropout rate in single-cell RNAseq expression data, only genes that exhibit high differential expression in both raw single-cell RNA sequencing data GSE118767 and microarray bulk RNA sequencing data GSE86337 are retained. The data is then normalized by library size and subjected to log-transformation before being fitted into the model. Finally, each cell expression vector is scaled to the range [0, 1] using the min-max normalization formula. Our simulation studies revealed that running the sc-*f*GAIN algorithm for 10,000 iterations provided sufficient training time for the generator and discriminator networks to converge to an optimal state. This convergence resulted in the generator producing more accurate imputations, while the discriminator effectively distinguished between real and generated data. Therefore, we consistently configured the model to run for 10,000 iterations in our experiments, varying the missing rate for the mask matrix. To ensure consistency with previous research, we adopted the same configuration as [34], while exploring different values for the hyperparameter of reconstruction loss. Additionally, we set the hint rate, which is used to generate the hint matrix from the mask vector, to 0.9 because our experiments have indicated that a hint rate of 0.9 will yield a smaller Root Mean Square Error (RMSE) for the missing value imputation. Previous studies [34] also showed that this hint rate yields the best performance in the naive GAIN model. The values of hyperparameters and sc-*f*GAIN code are released on the Github [42].

### Real data analysis

Next, we designed different experiments and used real scRNA-seq data with known ground truth values to evaluate the accuracy of sc-*f*GAIN’s imputation algorithm based on different *f*-divergence functions and compare with other imputation methods. In the first type of experiment, we intentionally introduced missing values into a portion of the data with varying rates of missingness to assess the effectiveness of sc-*f*GAIN in imputing missing values.

To validate the imputation accuracy of sc-*f*GAIN algorithm and other imputation methods, we computed the root-mean-square error (RMSE) between the true values and the imputed values in the scRNA-seq data, which has been used in many studies [34, 43]. The root mean squared error(RMSE) is defined as $RMSE=\sqrt{\frac{1}{n}\sum_{i}{\left(\hat{Y_{i}}-Y_{i}\right)}^{2}}$, where *Y*_*i*_ and $\hat{Y_{i}}$ represent true values and imputed values respectively.

#### sc-*f*GAIN can efficiently impute missing values

Most traditional imputation methods do not perform well when the cells are difficult to differentiate from one another. In order to achieve a high level of imputation accuracy for comparison purposes, we select a small subset of genes showing significant differential expression (DE) for the imputation process. Many methods [44–46] have been used to identify differentially expressed genes. Similar to [44], the Wilcoxon rank-sum test was used to identify differentially expressed genes, which was implemented through the Seurat package. Adjusted p-values were calculated using the Benjamini-Hochberg procedure which is a multiplicity correction method known for its ability to control the false discovery rate (FDR). Only those p-values < 0.001 in both single-cell and bulk RNAseq data are used for the first round data analysis, and finally 269 highly differentially expressed genes were selected. Table 3 reports the performance metric RMSE for various imputation methods given different missing rates for the A549 cell line. The lower the RMSE score, the better the imputation performance of the method.

**Table 3:** Comparison of RMSE scores for the imputation of 269 highly differentially expressed genes of the A549 cell line.

Missing rate	0.1	0.2	0.3	0.4	0.5
Method					
**MAGIC**	0.14219	0.16442	0.18861	0.21870	0.24963
**scImpute**	0.22818	0.23586	0.25137	0.26979	0.28257
**PBLR**	0.32017	0.32251	0.32208	0.32192	0.32085
**sc-*f*GAIN(CE)**	0.17808	0.18376	0.17894	0.17297	0.18989
**sc-*f*GAIN(FKL)**	0.20592	0.21683	0.20993	0.21219	0.20616
**sc-*f*GAIN(RKL)**	0.21701	0.21042	0.21238	0.20262	0.20425
**sc-*f*GAIN(JS)**	0.17589	0.18052	0.17499	0.17978	0.18176

Table 3’s results show that, the sc-*f*GAIN method outperformed most traditional imputation methods, with the *CE* and *JS* based sc-*f*GAIN method achieving the smallest RMSE score. Notably, our sc-*f*GAIN method exhibits remarkable robustness and minimal sensitivity to missing data rates, surpassing traditional imputation methods even under high missing data scenarios. The exceptional performance can be attributed to a fundamental advantage of our sc-fGAIN algorithm: it does not impose any assumptions about the underlying data distribution. This characteristic enhances the versatility of our approach, making it resilient against varying missing rates. Moreover, our method’s reliability is further underscored by rigorous theoretical analysis, confirming that the distribution of imputed data using the sc-fGAIN algorithm closely mirrors that of the original data. In comparsion, MAGIC only performed well when the missing rate was relatively low, but its performance deteriorated with higher dropout rates, moreover, PBLR and scImpute showed poor performance across all missing rates, and as the missing rate increases, all traditional methods’ imputation accuracy declines.

Furthermore, the results presented in Table 3 demonstrate that the performance of our sc-*f*GAIN algorithm is influenced by the selection of *f*-divergence functions. One major reason for the poor performance of traditional methods is the assumption that the missing values follow some specific distributions, which could not accurately capture the intricacy of the high-dimensional scRNA-seq data, and they rely on additional information about adjacent genes for constructing k-nearest neighbor graphs and clustering before imputation. Given that single-cell RNA sequencing data often exhibits a high dropout rate, our sc-*f*GAIN method outperforms many traditional methods and proves to be superior in handling scRNA-seq data. Table 4 presents the RMSE results for all cell lines. These results are consistent with the results reported for the single cell line in Table 3, that is, our sc-*f*GAIN method outperforms all the traditional methods when the missing rate is very high.

**Table 4:** Comparison of RMSE scores for the imputation of 269 highly differentially expressed genes of all cell lines.

Missing rate	0.1	0.2	0.3	0.4	0.5
Method					
**MAGIC**	0.13973	0.15795	0.17785	0.20032	0.21965
**scImpute**	0.31330	0.27651	0.26954	0.26381	0.26341
**PBLR**	0.27018	0.27012	0.26973	0.26985	0.26975
**sc-*f*GAIN(CE)**	0.16254	0.16486	0.16217	0.16387	0.16707
**sc-*f*GAIN(FKL)**	0.19953	0.20631	0.20816	0.21120	0.20968
**sc-*f*GAIN(RKL)**	0.24929	0.24818	0.23668	0.23782	0.22825
**sc-*f*GAIN(JS)**	0.16571	0.16299	0.16717	0.16326	0.16954

To investigate the impact of data size on the performance of the sc-*f*GAIN algorithm, we divided the entire dataset across genes into three equally sized parts and conducted imputation experiments on each split dataset. We also calculated the RMSE metric between the observed dataset and the combined imputed split data, which allows us to compare the results with those presented in Tables 3 and 4. The results in Table 5 demonstrate that the performance of the sc-*f*GAIN method is not very sensitive when the missing rate is low, but its performance will improve when the rate is high. Moreover, we observed that the sc-*f*GAIN method takes shorter running times to impute smaller split datasets. This observation presents an opportunity to leverage parallel computing and reduce imputation time by partitioning large datasets into smaller subsets across genes.

**Table 5:** RMSE score for the imputation of 269 DE genes on the split data.

Missing rate	0.1	0.2	0.3	0.4	0.5
Method					
**Results for all five cell lines**					
**sc-*f*GAIN(CE)**	0.13838	0.14969	0.15151	0.15596	0.16318
**sc-*f*GAIN(FKL)**	0.20000	0.19921	0.20915	0.20619	0.20478
**sc-*f*GAIN(RKL)**	0.22684	0.25093	0.23430	0.23482	0.22825
**sc-*f*GAIN(JS)**	0.13346	0.14648	0.14698	0.15795	0.15761
**Results for the A549 cell line**					
**sc-*f*GAIN(CE)**	0.11531	0.11917	0.12046	0.12025	0.12100
**sc-*f*GAIN(FKL)**	0.20050	0.21531	0.20917	0.20916	0.20836
**sc-*f*GAIN(RKL)**	0.21157	0.22077	0.21049	0.20686	0.20870
**sc-*f*GAIN(JS)**	0.11434	0.11850	0.11687	0.11714	0.12008

In another experiment, we included a larger dataset consisting of 2000 highly differentially expressed genes as input. These genes were selected using a similar approach as the work [25, 43]. The results in Table 6 align with those obtained from the small dataset presented in Tables 3 and 4. Both sc-*f*GAIN (FKL) and MAGIC methods achieves the lowest RMSE scores across all missing rates, and the other two sc-*f*GAIN methods based on CE and JS divergences remain competitive compared with the PBLR and scImpute, particularly when dealing with the data with high dropout rates. Both MAGIC and SAVER methods performed well when the missing rate was relatively low, but their performance deteriorated with higher dropout rates. MAGIC and sc-fGAIN are two competitive methods, their performance difference is small, this difference could be partially due to different normalization and rescaling approaches employed by MAGIC and sc-fGAIN, which can have an impact on how imputed values align with the original data. Our studies also found that, the sc-*f*GAIN method’s performance is influenced by the choice of loss function which is described by the *f*-divergence, as evident from the results in Tables 3–6. This indicates that sc-*f*GAIN offers a more comprehensive and versatile approach compared to the original GAIN model. To further explore the capabilities of sc-*f*GAIN, we have developed a convenient Huggingface tool. This tool, accessible at [47], provides users with the ability to identify the optimal *f*-divergence function for imputing a given dataset, leading to the lowest RMSE score. The corresponding code is hosted on GitHub at [48].

**Table 6:** Comparison of RMSE score (2000 genes) in the A549 cell line.

Missing rate	0.1	0.2	0.3	0.4	0.5
Method					
**MAGIC**	0.10309	0.09844	0.10168	0.11060	0.12283
**scImpute**	0.15627	0.15312	0.15401	0.15424	0.15479
**PBLR**	0.19374	0.19613	0.19655	0.19457	0.19055
**SAVER**	0.10545	0.13518	0.15233	0.16398	0.17242
**sc-*f*GAIN(CE)**	0.15206	0.15113	0.14939	0.15550	0.14953
**sc-*f*GAIN(FKL)**	0.12846	0.11287	0.11960	0.13058	0.12606
**sc-*f*GAIN(RKL)**	0.18133	0.17231	0.14557	0.14608	0.17078
**sc-*f*GAIN(JS)**	0.14958	0.15509	0.15329	0.15407	0.14922

Single-cell RNA sequencing data frequently encounters high dropout events, some traditional imputation methods make assumptions about the data distribution when filling in missing values. However, these assumptions can introduce bias and potentially lead to incorrect estimates of gene expression levels, thereby affecting downstream analysis. In contrast, the proposed sc-*f*GAIN method is a generative model that does not rely on any predefined data distribution assumptions. As a result, it is theoretically expected to mitigate the imputation bias compared to some traditional methods.

#### sc-*f*GAIN can reduce imputation variability

To assess the effectiveness of the imputation methods in reducing prediction variability, the gene-specific standard deviation (SD) across cells can be compared before and after imputation in the scRNA-seq data analysis. This standard deviation measures the inherent variability in gene expression for each gene across the single cells. A reduction in the standard deviation after imputation suggests that the imputation method has reduced prediction variability for the given gene, which also indicate that the imputed values have brought the data closer to a more stable or less noisy state. If the imputation method is not well-suited to the data or if it makes incorrect assumptions about the data distribution, there will be an increase in standard deviation after imputation.

**Fig 2:**
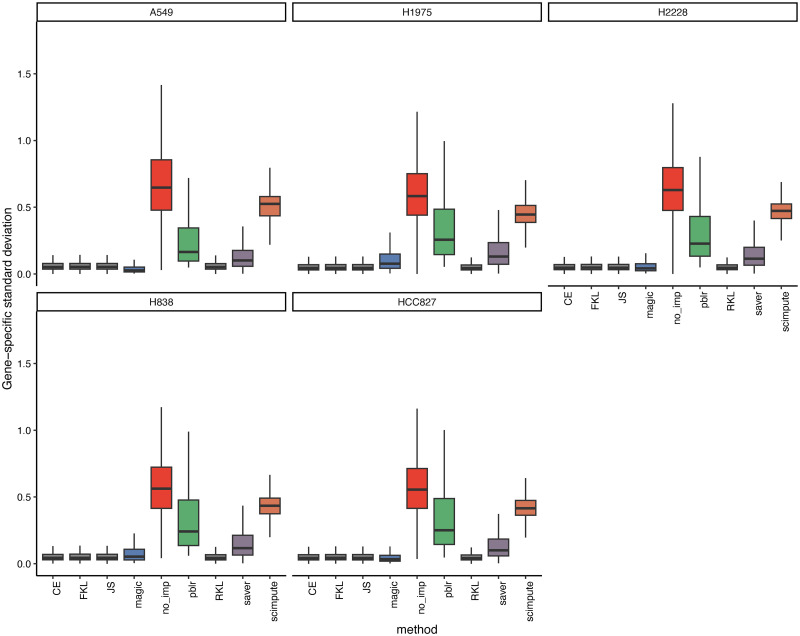
Comparison of sc-*f*GAIN imputation methods and traditional methods in terms of gene-specific standard deviation. The x-axis represents different imputation methods, and y-axis represents the gene-specific standard deviation.

Fig 2 demonstrates the changes of the standard deviation (SD) across different imputation methods in each cell line. Prior to imputation (red box), the SD values range from 0.1 to 1.4, with a median value exceeding 0.5. However, when applying the traditional imputation methods such as scImpute (brown box) and PBLR (green box), there is only a slight reduction in the SD values, which range from 0.1 to 1.0 with a median value around 0.3–0.5. It is important to note that a higher standard deviation indicates higher uncertainty or variability in the imputed values, reflecting the limitations of these two imputation methods. The relatively small reduction in SD values suggests that scImpute and PBLR are not able to effectively address the uncertainty and variability associated with missing values in the dataset. The poor performance of PBLR can be attributed to the assumption made regarding the prior distribution of the missing values, which may not accurately represent the true underlying distribution of the missing values. On the other hand, scImpute’s high prediction variability can be attributed to its assumption that the underlying gene expression data matrix has a low-rank structure, that is, the majority of the genetic information can be captured by a smaller number of latent factors. However, the scRNA-seq data exhibit more intricate patterns and dependencies that might not be adequately represented by a low-rank structure. These assumptions limit the ability of PBLR and scImpute to capture the complexity of the data, leading to a large imputation variability.

In contrast, our sc-*f*GAIN methods, by leveraging generative adversarial networks and utilizing different *f*-divergences (CE, FKL, RKL, JS), overcome the limitations imposed by the assumptions made in traditional imputation methods. When using the sc-*f*GAIN methods, the resulting standard deviation values are consistently lower compared to traditional imputation methods. All the median values are lower than 0.1 with small variations, which indicates a significant decrease in the variability of the imputed values compared to the initial missing values. Among the traditional methods, only MAGIC demonstrates comparable performance to our sc-*f*GAIN method. However, it is important to note that our sc-*f*GAIN method offers flexibility in choosing different *f*-divergences, allowing for better adaptation to diverse data patterns. This finding suggests that imputation using sc-*f*GAIN reduce the imputation variability compared to traditional methods. Consequently, sc-*f*GAIN enables accurate imputation of missing expression values without significantly altering the overall variability of the data, thereby facilitating improved downstream analyses of single-cell RNA sequencing data.

## Conclusion

In this work, we have developed a novel *f*-divergence based generative adversarial imputation network method, called sc-*f*GAIN, to impute missing values in single-cell RNA sequencing data. Unlike some traditional imputation methods, sc-*f*GAIN employs a generative model to generate synthetic values without making any distribution assumptions, the missing values are imputed by training two neural networks whose objective functions are described by *f*-divergence. Our theoretical studies, for the first time, identified only four *f*-divergences, namely cross-entropy, forward Kullback-Leibler (KL), reverse KL, and Jensen-Shannon, that can be effectively used as loss functions to train the sc-*f*GAIN algorithm to generate imputed values without any assumptions, and mathematically proved that the distribution of imputed data using sc-*f*GAIN algorithm is same as the distribution of original data.

Our experiments with public scRNA-seq data have shown that the proposed sc-*f*GAIN method has many advantages compared with some traditional imputation methods and vanilla GAIN. Specifically, the imputed values generated by sc-*f*GAIN have a smaller root-mean-square error compared to many traditional methods. Importantly, the performance of sc-*f*GAIN is robust to varying missing rates of scRNA-seq data, whereas most traditional methods’ performance will deteriorate with higher missing rates. The studies also indicate that the performance of sc-*f*GAIN is sensitive to the choice of *f*-divergence used in the loss function. So, the proposed sc-*f*GAIN algorithm is more comprehensive and versatile compared to the naive GAIN model. Our tool can also help to identify a suitable *f*-divergence function to optimize the performance of the sc-*f*GAIN algorithm. Moreover, our investigation also revealed that the sc-*f*GAIN method can reduce imputation variability by producing smaller standard deviations of gene expression values compared to most traditional methods. This indicates that sc-*f*GAIN is capable of accurately imputing missing values without altering the overall variability of the data. Such an ability can help improve downstream analyses that rely on accurate estimation of gene expression levels.

Despite its promising potential, sc-*f*GAIN has some limitations that should be acknowledged. One such limitation is its training speed, particularly when working with large datasets. For instance, analyzing the scRNAseq dataset GSE118767 using sc-*f*GAIN can take approximately 3 hours on a Tesla V100-SXM2 16GB GPU. However, there are several approaches that can be implemented to mitigate this issue, including reducing the batch size, minimizing the number of iterations, and partitioning the dataset into smaller subsets for separate imputations. Our investigations have revealed that employing these strategies does not significantly compromise the accuracy of the results, making them feasible solutions for addressing the training speed limitation. Another limitation we acknowledge is our inability to distinguish between biological zeros (genes that were genuinely not expressed during sequencing) and technical zeros (genes that were expressed during sequencing but not accurately measured), which has been investigated using a low-rank matrix approximation method [49] recently. We plan to explore strategies to enhance our algorithm’s capability to impute technical zeros while preserving biological zeros in our future studies. In conclusion, our findings highlight the promising potential of sc-*f*GAIN in enhancing the quality of single-cell RNA sequencing data and facilitating more precise and reliable downstream analyses.

## Supporting information

S1 Appendix Proof of Theorem 1-2. Detailed proof of the Theorem 1-2 in the Section: Theoretical analysis of sc-*f*GAIN Algorithm.
(PDF)

## References

[1] Yanai I, Hashimshony T. CEL-Seq2-Single-cell RNA sequencing by multiplexed linear amplification. Single Cell Methods: Sequencing and Proteomics. 2019; p. 45–56. doi: 10.1007/978-1-4939-9240-9_4 31028631

[2] Zheng GX, Terry JM, Belgrader P, Ryvkin P, Bent ZW, Wilson R, et al. Massively parallel digital transcriptional profiling of single cells. Nature communications. 2017;8(1):14049. doi: 10.1038/ncomms14049 28091601 PMC5241818

[3] Velmeshev D, Schirmer L, Jung D, Haeussler M, Perez Y, Mayer S, et al. Single-cell genomics identifies cell type–specific molecular changes in autism. Science. 2019;364(6441):685–689. doi: 10.1126/science.aav8130 31097668 PMC7678724

[4] Imoto S, Goto T, Miyano S. Estimation of genetic networks and functional structures between genes by using BN and nonparametric regression. Pacific symposium on Biocomputing. 2002; 175–86. 11928473

[5] Kim S, Imoto S, Miyano S. Inferring gene networks from time series microarray data using dynamic Bayesian networks. Briefings in Bioinformatics. 2003;4:228–235. doi: 10.1093/bib/4.3.228 14582517

[6] Friedman N, Murphy K, Russell S. Learning the Structure of Dynamic Probabilistic Networks. In: Proceedings of the Fourteenth Conference on Uncertainty in Artificial Intelligence. Morgan Kaufmann Publishers Inc.; 1998. p. 139–147.

[7] Ong I, Glasner J, Page D. Modelling regulatory pathways in E. coli from time series expression profiles. Bioinformatics. 2002;18:S241–S248. doi: 10.1093/bioinformatics/18.suppl_1.S241 12169553

[8] Kim S, Imoto S, Miyano S. Dynamic Bayesian network and nonparametric regression for nonlinear modeling of gene networks from time series gene expression data. BioSystems. 2004;75:57–65. doi: 10.1016/j.biosystems.2004.03.004 15245804

[9] Richards H, Wang Y, Si T, Zhang H, Gong H. Intelligent Learning and Verification of Biological Networks. Advances in Artificial Intelligence, Computation, and Data Science: For Medicine and Life Science. 2021; p. 3–28.

[10] Molinari C, Marisi G, Passardi A, Matteucci L, De Maio G, Ulivi P. Heterogeneity in colorectal cancer: a challenge for personalized medicine? International journal of molecular sciences. 2018;19(12):3733. doi: 10.3390/ijms19123733 30477151 PMC6321493

[11] Shapiro E, Biezuner T, Linnarsson S. Single-cell sequencing-based technologies will revolutionize whole-organism science. Nature Reviews Genetics. 2013;14(9):618–630. doi: 10.1038/nrg3542 23897237

[12] Hedlund E, Deng Q. Single-cell RNA sequencing: technical advancements and biological applications. Molecular aspects of medicine. 2018;59:36–46. doi: 10.1016/j.mam.2017.07.003 28754496

[13] Bates S. Progress towards personalized medicine. Drug discovery today. 2010;15(3-4):115–120. doi: 10.1016/j.drudis.2009.11.001 19914397

[14] Li WV, Li JJ. An accurate and robust imputation method scImpute for single-cell RNA-seq data. Nature communications. 2018;9(1):1–9. doi: 10.1038/s41467-018-03405-7 29520097 PMC5843666

[15] Vieth B, Parekh S, Ziegenhain C, Enard W, Hellmann I. A systematic evaluation of single cell RNA-seq analysis pipelines. Nature communications. 2019;10(1):4667. doi: 10.1038/s41467-019-12266-7 31604912 PMC6789098

[16] Saliba AE, Westermann AJ, Gorski SA, Vogel J. Single-cell RNA-seq: advances and future challenges. Nucleic acids research. 2014;42(14):8845–8860. doi: 10.1093/nar/gku555 25053837 PMC4132710

[17] Lähnemann D, Köster J, Szczurek E, McCarthy DJ, Hicks SC, Robinson MD, et al. Eleven grand challenges in single-cell data science. Genome biology. 2020;21(1):1–35. doi: 10.1186/s13059-020-1926-6 32033589 PMC7007675

[18] Villani AC, Satija R, Reynolds G, Sarkizova S, Shekhar K, Fletcher J, et al. Single-cell RNA-seq reveals new types of human blood dendritic cells, monocytes, and progenitors. Science. 2017;356(6335):eaah4573. doi: 10.1126/science.aah4573 28428369 PMC5775029

[19] Zhang L, Zhang S. Comparison of computational methods for imputing single-cell RNA-sequencing data. IEEE/ACM transactions on computational biology and bioinformatics. 2018;17(2):376–389. 29994128 10.1109/TCBB.2018.2848633

[20] Chen M, Zhou X. VIPER: variability-preserving imputation for accurate gene expression recovery in single-cell RNA sequencing studies. Genome biology. 2018;19(1):1–15. doi: 10.1186/s13059-018-1575-1 30419955 PMC6233584

[21] van Dijk D, Nainys J, Sharma R, Kaithail P, Carr AJ, Moon KR, et al. MAGIC: A diffusion-based imputation method reveals gene-gene interactions in single-cell RNA-sequencing data. BioRxiv. 2017; p. 111591.

[22] Huang M, Wang J, Torre E, Dueck H, Shaffer S, Bonasio R, et al. SAVER: gene expression recovery for single-cell RNA sequencing. Nature methods. 2018;15(7):539–542. doi: 10.1038/s41592-018-0033-z 29941873 PMC6030502

[23] Zhang L, Zhang S. PBLR: an accurate single cell RNA-seq data imputation tool considering cell heterogeneity and prior expression level of dropouts. bioRxiv. 2018; p. 379883.

[24] Arisdakessian C, Poirion O, Yunits B, Zhu X, Garmire LX. DeepImpute: an accurate, fast, and scalable deep neural network method to impute single-cell RNA-seq data. Genome biology. 2019;20(1):1–14. doi: 10.1186/s13059-019-1837-6 31627739 PMC6798445

[25] Wang J, Ma A, Chang Y, Gong J, Jiang Y, Qi R, et al. scGNN is a novel graph neural network framework for single-cell RNA-Seq analyses. Nature communications. 2021;12(1):1882. doi: 10.1038/s41467-021-22197-x 33767197 PMC7994447

[26] Hou W, Ji Z, Ji H, Hicks SC. A systematic evaluation of single-cell RNA-sequencing imputation methods. Genome biology. 2020;21:1–30. doi: 10.1186/s13059-020-02132-x 32854757 PMC7450705

[27] Xu J, Cui L, Zhuang J, Meng Y, Bing P, He B, et al. Evaluating the performance of dropout imputation and clustering methods for single-cell RNA sequencing data. Computers in Biology and Medicine. 2022; p. 105697. doi: 10.1016/j.compbiomed.2022.105697 35697529

[28] Wagner F, Yan Y, Yanai I. K-nearest neighbor smoothing for high-throughput single-cell RNA-Seq data. BioRxiv. 2017; p. 217737.

[29] Klebanov L, Yakovlev A. Diverse correlation structures in gene expression data and their utility in improving statistical inference. The Annals of Applied Statistics. 2007;1:538–559. doi: 10.1214/07-AOAS120

[30] Goodfellow I, Pouget-Abadie J, Mirza M, Xu B, Warde-Farley D, Ozair S, et al. Generative adversarial networks. Communications of the ACM. 2020;63(11):139–144. doi: 10.1145/3422622

[31] Arjovsky M, Chintala S, Bottou L. Wasserstein generative adversarial networks. In: International conference on machine learning. PMLR; 2017. p. 214–223.

[32] Gulrajani I, Ahmed F, Arjovsky M, Dumoulin V, Courville AC. Improved training of wasserstein gans. Advances in neural information processing systems. 2017;30.

[33] Li Y, Min M, Shen D, Carlson D, Carin L. Video generation from text. In: Proceedings of the AAAI conference on artificial intelligence. vol. 32; 2018.

[34] Yoon J, Jordon J, Schaar M. Gain: Missing data imputation using generative adversarial nets. In: International conference on machine learning. PMLR; 2018. p. 5689–5698.

[35] Kurach K, Lučić M, Zhai X, Michalski M, Gelly S. A Large-Scale Study on Regularization and Normalization in GANs. In: Chaudhuri K, Salakhutdinov R, editors. Proceedings of the 36th International Conference on Machine Learning. vol. 97 of Proceedings of Machine Learning Research. PMLR; 2019. p. 3581–3590.

[36] Nowozin S, Cseke B, Tomioka R. f-gan: Training generative neural samplers using variational divergence minimization. Advances in neural information processing systems. 2016;29.

[37] Ali SM, Silvey SD. A general class of coefficients of divergence of one distribution from another. Journal of the Royal Statistical Society: Series B (Methodological). 1966;28(1):131–142.

[38] Hiriart-Urruty JB, Lemaréchal C. Fundamentals of convex analysis. Springer Science & Business Media; 2004.

[39] Tian L, Dong X, Freytag S, Lê Cao KA, Su S, JalalAbadi A, et al. Benchmarking single cell RNA-sequencing analysis pipelines using mixture control experiments. Nature methods. 2019;16(6):479–487. doi: 10.1038/s41592-019-0425-8 31133762

[40] Wang X, He Y, Zhang Q, Ren X, Zhang Z. Direct comparative analyses of 10X genomics chromium and smart-seq2. Genomics, proteomics & bioinformatics. 2021;19(2):253–266. doi: 10.1016/j.gpb.2020.02.005 33662621 PMC8602399

[41] Holik AZ, Law CW, Liu R, Wang Z, Wang W, Ahn J, et al. RNA-seq mixology: designing realistic control experiments to compare protocols and analysis methods. Nucleic acids research. 2017;45(5):e30–e30. doi: 10.1093/nar/gkw1063 27899618 PMC5389713

[42] Computer code. Available from: https://github.com/TongSii/sc-fGAIN.

[43] Mera-Gaona M, Neumann U, Vargas-Canas R, López DM. Evaluating the impact of multivariate imputation by MICE in feature selection. Plos one. 2021;16(7):e0254720. doi: 10.1371/journal.pone.0254720 34320016 PMC8318311

[44] Yang X, Zhu S, Li L, Zhang L, Xian S, Wang Y, et al. Identification of differentially expressed genes and signaling pathways in ovarian cancer by integrated bioinformatics analysis. OncoTargets and therapy. 2018; p. 1457–1474. doi: 10.2147/OTT.S152238 29588600 PMC5858852

[45] Liu ZK, Zhang RY, Yong YL, Zhang ZY, Li C, Chen ZN, et al. Identification of crucial genes based on expression profiles of hepatocellular carcinomas by bioinformatics analysis. PeerJ. 2019;7:e7436. doi: 10.7717/peerj.7436 31410310 PMC6689388

[46] Zhao B, Erwin A, Xue B. How many differentially expressed genes: a perspective from the comparison of genotypic and phenotypic distances. Genomics. 2018;110(1):67–73. doi: 10.1016/j.ygeno.2017.08.007 28843784

[47] Huggingface Tool. Available from: https://huggingface.co/spaces/zhopkins/fGAIN.

[48] Huggingface code. Available from: https://github.com/TongSii/hugging-face-demo.

[49] Linderman G, Zhao J, Roulis M, Bielecki P, Flavell R, Nadler B, et al. Zero-preserving imputation of single-cell RNA-seq data. Nature communications. 2022; 13(1):192. doi: 10.1038/s41467-021-27729-z 35017482 PMC8752663

