# Supplementary material for: A novel f-divergence based generative adversarial imputation method for scRNA-seq data analysis
Source: PLoS One. 2023 Nov 10;18(11):e0292792. doi: 10.1371/journal.pone.0292792 (PMC10637660; doi:10.1371/journal.pone.0292792)
Supplement: S1 Appendix — Detailed proof of the Theorem 1-2 in the Section: Theoretical analysis of sc-fGAIN Algorithm. (PDF) [file pone.0292792.s001.pdf]

S1 Appendix: Proof of Theorem 1-2

Proof of Theorem 1

We will use the following equation,

$$\frac{\partial}{\partial g_f(S_\phi)_i} f^*(g_f(S_\phi))_i = \frac{p(\mathbf{x}, \mathbf{h}, m_i = 1)}{p(\mathbf{x}, \mathbf{h}, m_i = 0)}. \tag{1}$$

and Table 2 to prove the Theorem 1.
Set $p_i = p(\mathbf{x}, \mathbf{h}, m_i = 1)$, $q_i = p(\mathbf{x}, \mathbf{h}, m_i = 0)$, apply the Sigmoid function on the output of the discriminator network $S_\phi(x, h)$, that is $D_i = \frac{1}{1+\exp(-S_\phi(x,h)_i)}$.

Below, we will derive the optimal discriminator D_i^* given different f-divergence functions:

- f = **Cross-entropy (CE):**

$$\frac{\partial}{\partial g_f(S_\phi)_i} f^*(g_f(S_\phi))_i = \frac{p_i}{q_i} = \frac{\exp(-\log(1+\exp(-S_\phi(x,h)_i))}{1-\exp(-\log(1+\exp(-S_\phi(x,h)_i))} = \frac{\frac{1}{1+\exp(-S_\phi(x,h)_i)}}{1-\frac{1}{1+\exp(-S_\phi(x,h)_i)}}$$

  The above equation can be simplified as $\frac{p_i}{q_i} = \frac{D_i}{1-D_i}$.
  Finally, we get the optimal discriminator $D_i^* = \frac{p_i}{p_i+q_i}$.

- f = **Kullback-Leibler(FKL)**
  $\frac{\partial}{\partial g_f(S_\phi)_i} f^*(g_f(S_\phi))_i = \frac{p_i}{q_i} = \exp(-\log(\frac{1}{S_\phi(x,h)_i} - 1) - 1)$
  The above equation can be simplified as $\frac{p_i}{q_i} = \frac{D_i}{e(1-D_i)}$.
  Finally, we get the optimal discriminator $D_i^* = \frac{p_i e}{p_i e + q_i}$.

- f = **Reverse Kullback-Leibler (RKL)**
  $\frac{\partial}{\partial g_f(S_\phi)_i} f^*(g_f(S_\phi))_i = \frac{p_i}{q_i} = \frac{1}{-\exp(-S_\phi(x,h)_i)}$
  The above equation can be simplified as $\frac{p_i}{q_i} = \frac{D_i}{1-D_i}$.
  Finally, we get the optimal discriminator $D_i^* = \frac{p_i}{p_i+q_i}$

- f = **Jensen-Shannon (JS)**
  $\frac{\partial}{\partial g_f(S_\phi)_i} f^*(g_f(S_\phi))_i = \frac{p_i}{q_i} = \frac{\frac{2}{1+\exp(-S_\phi(x,h)_i)}}{2-\frac{2}{1+\exp(-S_\phi(x,h)_i)}}$
  The above equation can be simplified as $\frac{p_i}{q_i} = \frac{D_i}{1-D_i}$.
  Finally, we get the optimal discriminator $D_i^* = \frac{p_i}{p_i+q_i}$.

- f = **Pearson χ^2 (PC)**
  $\frac{\partial}{\partial g_f(S_\phi)_i} f^*(g_f(S_\phi))_i = \frac{p}{q} = \frac{1}{2} S_\phi(x,h)_i + 1$
  Finally, we get the optimal discriminator $D_i^* = \frac{exp(2(p_i-q_i)/q_i)}{1+exp(2(p_i-q_i)/q_i)}$.

Proof of Theorem 2

Given the optimal discriminator D_i^*, for each f-divergence function, the objective function $\mathcal{L}_{G,f}(D^*)$ can be expressed as:

- f = **CE**

$$\begin{aligned}\mathcal{L}_{G,f}(D^*) &= \sum_{i}^{d}\int_{\mathcal{X}}\int_{\mathcal{H}}(p_i \log(D_i^*) + q_i \log(1 - D_i^*))\, dxdh \\ &= \sum_{i}^{d}\int_{\mathcal{X}}\int_{\mathcal{H}}\left(p_i \log(\frac{p_i}{p_i + q_i}) + q_i \log(\frac{q_i}{p_i + q_i})\right) dxdh\end{aligned}$$

It can also be written as:

$$\begin{aligned}\mathcal{L}_{G,f}(D^*) &= \sum_{t\in\{0,1\}}\sum_{i=1}^{d}\int_{\mathcal{X}}\int_{\mathcal{H}} p(\mathbf{x},\mathbf{h},m_i=t)\log(\frac{p(\mathbf{x},\mathbf{h},m_i=t)}{p(\mathbf{x},\mathbf{h},m_i=0)+p(\mathbf{x},\mathbf{h},m_i=1)})dhdx \\ &= \sum_{t\in\{0,1\}}\sum_{i=1}^{d}\int_{\mathcal{X}}\int_{\mathcal{H}} p(\mathbf{x},\mathbf{h},m_i=t)\log(\frac{p(\mathbf{x},m_i=t|\mathbf{h})p(m_i=t|h)}{p(\mathbf{x}|\mathbf{h})p(m_i=t|h)})dhdx \\ &= \sum_{t\in\{0,1\}}\sum_{i=1}^{d}\int_{\mathcal{X}}\int_{\mathcal{H}} p(\mathbf{h},m_i=t)p(\mathbf{x}|\mathbf{h},m_i=t)\log(\frac{p(\mathbf{x}|\mathbf{h},m_i=t)}{p(\mathbf{x}|\mathbf{h}))}dhdx \\ &+ \sum_{t\in\{0,1\}}\sum_{i=1}^{d}\int_{\mathcal{H}} p(\mathbf{h},m_i=t)\log(p(m_i=t|\mathbf{h}))dh \\ &= \sum_{t\in\{0,1\}}\sum_{i=1}^{d}\int_{\mathcal{H}} p(\mathbf{h},m_i=t)D_{KL}(p(x|\mathbf{h},m_i=t)||p(x|\mathbf{h}))dh \\ &+ \sum_{t\in\{0,1\}}\sum_{i=1}^{d}\int_{\mathcal{H}} p(\mathbf{h},m_i=t)\log(p(m_i=t|\mathbf{h}))dh\end{aligned}$$

Since KL divergence is non-negative, so the loss function $\mathcal{L}_{G,f}(D^*)$ is minimized if and only if $\hat{p}(\mathbf{x}|\mathbf{h},m_i=t)=\hat{p}(\mathbf{x}|\mathbf{h})$ for any $i\in\{1,...,d\}$.

- f = **FKL**

$$
\begin{aligned}
\mathcal{L}_{G,f}(D^*) &= \sum_{i=1}^{d} \int_{\chi} \int_{\mathcal{H}} \left(-p(\frac{1}{D^*} - 1) + q(1 + \log(\frac{1}{D^*} - 1)) \right) dxdh \\
&= \sum_{i=1}^{d} \int_{\chi} \int_{\mathcal{H}} \left(p \log \frac{p}{q} \right) dxdh \\
&= \sum_{i=1}^{d} \int_{\chi} \int_{\mathcal{H}} \left(p \log p + q \log q - (p+q) \log q \right) dxdh \\
&= \sum_{i=1}^{d} \int_{\chi} \int_{\mathcal{H}} \sum_{t \in \{0,1\}} p(\mathbf{x}, \mathbf{h}, m_i = t) \log \frac{P(x|h, m_i = t)}{P(x|h)} dxdh \\
&+ \sum_{t \in \{0,1\}} \sum_{i=1}^{d} \int_{\mathcal{H}} p(\mathbf{h}, m_i = t) \log(p(m_i = t|\mathbf{h})) dh \\
&- \sum_{i=1}^{d} \int_{\chi} \int_{\mathcal{H}} p(x,h) \log p(m_i = 0|x,h) dxdh \\
&= \sum_{i=1}^{d} \int_{\chi} \int_{\mathcal{H}} \sum_{t \in \{0,1\}} p(\mathbf{x}|\mathbf{h}, m_i = t) p(\mathbf{h}, m_i = t) \log \frac{P(x|h, m_i = t)}{P(x|h)} dxdh \\
&+ \sum_{t \in \{0,1\}} \sum_{i=1}^{d} \int_{\mathcal{H}} p(\mathbf{h}, m_i = t) \log(p(m_i = t|\mathbf{h})) dh \\
&- \sum_{i=1}^{d} \int_{\chi} \int_{\mathcal{H}} p(h) p(x|h) \log \frac{p(x, m_i = 0|h)}{p(x|h)} dxdh \\
&= \sum_{t \in \{0,1\}} \sum_{i=1}^{d} \int_{\mathcal{H}} p(\mathbf{h}, m_i = t) D_{KL}(p(x|\mathbf{h}, m_i = t) || p(x|\mathbf{h})) \\
&+ p(\mathbf{h}, m_i = t) \log(p_m(m_i = t|\mathbf{h})) dh \\
&+ \sum_{i=1}^{d} \int_{\mathcal{H}} p(h) D_{KL}(p(x|h) || p(x|m_i = 0, h)) - p(h) \log p(m_i = 0|h) dh
\end{aligned}
$$

Similar to CE's proof, since KL divergence is non-negative, so $\mathcal{L}_{G,f}(D^*)$ is minimized if and only if $p(x|\mathbf{h}, m_i = t) = p(x|\mathbf{h})$ for the 1st and 2nd term.

- f **= RKL**

$$\begin{aligned}
\mathcal{L}_{G,f}(D^*) &= \sum_{i=1}^{d}\int_{\chi}\int_{\mathcal{H}}\left(p\log(\frac{D^*}{1-D^*}) - \frac{q}{e}\frac{D^*}{1-D^*}\right)dxdh \\
&= \sum_{i=1}^{d}\int_{\chi}\int_{\mathcal{H}}\left(p\log(\frac{pe}{q}) - \frac{q}{e}\log(\frac{pe}{q})\right)dxdh = \sum_{i=1}^{d}\int_{\chi}\int_{\mathcal{H}}\left(q\log\frac{q}{p}\right)dxdh \\
&= \sum_{i=1}^{d}\int_{\chi}\int_{\mathcal{H}}\left(q\log q + p\log p - (p+q)\log(p)\right)dxdh \\
&= \sum_{i=1}^{d}\int_{\chi}\int_{\mathcal{H}}\sum_{t\in\{0,1\}} p(\mathbf{x},\mathbf{h},m_i=t)\log\frac{P(x|h,m_i=t)}{P(x|h)}dxdh \\
&+ \sum_{t\in\{0,1\}}\sum_{i=1}^{d}\int_{\mathcal{H}} p(\mathbf{h},m_i=t)\log(p(m_i=t|\mathbf{h}))dh \\
&- \sum_{i=1}^{d}\int_{\chi}\int_{\mathcal{H}} p(x,h)\log p(m_i=1|x,h)dxdh \\
&= \sum_{i=1}^{d}\int_{\chi}\int_{\mathcal{H}}\sum_{t\in\{0,1\}} p(\mathbf{x}|\mathbf{h},m_i=t)p(\mathbf{h},m_i=t)\log\frac{P(x|h,m_i=t)}{P(x|h)}dxdh \\
&+ \sum_{t\in\{0,1\}}\sum_{i=1}^{d}\int_{\mathcal{H}} p(\mathbf{h},m_i=t)\log(p(m_i=t|\mathbf{h}))dh \\
&- \sum_{i=1}^{d}\int_{\chi}\int_{\mathcal{H}} p(h)p(x|h)\log\frac{p(x,m_i=1|h)}{p(x|h)}dxdh \\
&= \sum_{t\in\{0,1\}}\sum_{i=1}^{d}\int_{\mathcal{H}} p(\mathbf{h},m_i=t)D_{KL}(p(x|\mathbf{h},m_i=t)||p(x|\mathbf{h})) \\
&+ p(\mathbf{h},m_i=t)\log(p_m(m_i=t|\mathbf{h}))dh \\
&+ \sum_{i=1}^{d}\int_{\mathcal{H}} p(h)D_{KL}(p(x|h)||p(x|m_i=1,h)) - p(h)\log p(m_i=1|h)dh
\end{aligned}$$

Similarly to FKL, since KL divergence is non-negative, so, to get $\mathcal{L}_{G,f}(D^*)$ minimized if and only if $p(x|\mathbf{h},m_i=t) = p(x|\mathbf{h})$.

- f = **JS**

$$
\begin{aligned}
\mathcal{L}_{G,f}(D^*) &= \sum_{t\in\{0,1\}} \sum_{i=1}^{d} \int_{\mathcal{X}} \int_{\mathcal{H}} \log(2D^*) + q\log(2-2D^*) \\
&= \sum_{t\in\{0,1\}} \sum_{i=1}^{d} \int_{\mathcal{X}} \int_{\mathcal{H}} p\log\left(\frac{2p}{p+q}\right) + q\log\left(\frac{2q}{p+q}\right) \\
&= \sum_{t\in\{0,1\}} \sum_{i=1}^{d} \int_{\mathcal{X}} \int_{\mathcal{H}} p(\mathbf{x},\mathbf{h},m_i=t)\log\left(\frac{p(\mathbf{x},\mathbf{h},m_i=t)}{p(\mathbf{x},\mathbf{h},m_i=0)+p(\mathbf{x},\mathbf{h},m_i=1)}\right)dhdx \\
&+ \sum_{t\in\{0,1\}} \sum_{i=1}^{d} \int_{\mathcal{X}} \int_{\mathcal{H}} p(\mathbf{x},\mathbf{h},m_i=t)\log(2)dxdh \\
&= \sum_{t\in\{0,1\}} \sum_{i=1}^{d} \int_{\mathcal{X}} \int_{\mathcal{H}} p(\mathbf{x},\mathbf{h},m_i=t)\log\left(\frac{p(\mathbf{x},m_i=t|\mathbf{h})p_m(m_i=t|h)}{p(\mathbf{x}|\mathbf{h})p_m(m_i=t|h)}\right)dhdx + C \\
&= \sum_{t\in\{0,1\}} \sum_{i=1}^{d} \int_{\mathcal{X}} \int_{\mathcal{H}} p(\mathbf{h},m_i=t)p(\mathbf{x}|\mathbf{h},m_i=t)\log\left(\frac{p(\mathbf{x}|\mathbf{h},m_i=t)}{p(\mathbf{x}|\mathbf{h})}\right)dhdx \\
&+ \sum_{t\in\{0,1\}} \sum_{i=1}^{d} \int_{\mathcal{H}} p(\mathbf{h},m_i=t)p(\mathbf{x}|\mathbf{h},m_i=t)\log(p_m(m_i=t|\mathbf{h}))dh + C \\
&= \sum_{t\in\{0,1\}} \sum_{i=1}^{d} \int_{\mathcal{H}} p(\mathbf{h},m_i=t)D_{KL}(p(\cdot|\mathbf{h},m_i=t)||p(\cdot|\mathbf{h}))dh \\
&+ \sum_{t\in\{0,1\}} \sum_{i=1}^{d} \int_{\mathcal{H}} p(\mathbf{h},m_i=t)\log(p_m(m_i=t|\mathbf{h}))dh + C
\end{aligned}
$$

C denotes a constant, therefore, the loss function is minimized if $D_{KL}(p||q)=0$, that is, $p(x|\mathbf{h},m_i=t)=p(x|\mathbf{h})$
